# Protein-, (Poly)peptide-, and Amino Acid-Based Nanostructures Prepared via Polymerization-Induced Self-Assembly

**DOI:** 10.3390/polym13162603

**Published:** 2021-08-05

**Authors:** Spyridon Varlas, Georgia L. Maitland, Matthew J. Derry

**Affiliations:** 1 Department of Chemistry, University College London, London WC1H 0AJ, UK; 2 Aston Institute of Materials Research, Aston University, Birmingham B4 7ET, UK; maitlang@aston.ac.uk

**Keywords:** proteins, polypeptides, α-amino acids, block copolymers, polymerization-induced self-assembly, protein–polymer conjugates, nanostructures, biomaterials

## Abstract

Proteins and peptides, built from precisely defined amino acid sequences, are an important class of biomolecules that play a vital role in most biological functions. Preparation of nanostructures through functionalization of natural, hydrophilic proteins/peptides with synthetic polymers or upon self-assembly of all-synthetic amphiphilic copolypept(o)ides and amino acid-containing polymers enables access to novel protein-mimicking biomaterials with superior physicochemical properties and immense biorelevant scope. In recent years, polymerization-induced self-assembly (PISA) has been established as an efficient and versatile alternative method to existing self-assembly procedures for the reproducible development of block copolymer nano-objects in situ at high concentrations and, thus, provides an ideal platform for engineering protein-inspired nanomaterials. In this review article, the different strategies employed for direct construction of protein-, (poly)peptide-, and amino acid-based nanostructures via PISA are described with particular focus on the characteristics of the developed block copolymer assemblies, as well as their utilization in various pharmaceutical and biomedical applications.

## 1. Introduction

Proteins and peptides are essential components in nature’s toolkit, since they are responsible for the proper function, structural organization, and protection of cells, organs and tissues. Their critical role in regulating the majority of biochemical processes, as well as the transport and storage of nutrients and other signaling molecules within living organisms [1,2], has directed a significant part of scientific research toward understanding, utilizing, and mimicking these biomacromolecules for various biomedical applications, including nanomedicine [3,4,5], biocatalysis [6,7], enzyme-mediated therapy [8,9], and tissue engineering [10,11]. Nonetheless, proteins and natural peptides often exhibit poor stability, reduced circulation times in vivo and increased immunogenicity, considerably limiting their applicability [12,13]. A common strategy employed to improve their biophysical properties involves their functionalization with synthetic polymers for preparation of protein/peptide–polymer conjugates and hybrid nano-assemblies [14,15,16,17]. Additionally, alternative approaches for the development of advanced protein-inspired nanomaterials encompass either the synthesis of amphiphilic polypeptide- and polypeptoid-based copolymers via ring-opening polymerization (ROP) of α-amino acid *N*-carboxyanhydrides (NCAs) [18,19,20] or the synthesis of amino acid-containing amphiphiles via controlled radical polymerization techniques [21,22], followed by their self-assembly in aqueous media to yield biorelevant nanostructures of varying morphologies. However, until recently, efforts to develop such self-assembled formulations have majorly focused on strenuous, multi-step procedures at low polymer concentrations, which are often poorly reproducible.

Over the past decade, polymerization-induced self-assembly (PISA) has emerged as a powerful method for the simultaneous synthesis and solution self-assembly of block copolymers in a range of media [23,24,25]. In contrast to traditional post-polymerization self-assembly techniques, such as solvent-switch or thin-film rehydration, PISA enables the in situ formation of block copolymer nano-objects at high solids contents (up to 50% *w*/*w*) [26]. Briefly, PISA involves the chain-extension of a functional solvophilic polymer with a range of solvent-miscible or solvent-immiscible monomers to yield amphiphilic block copolymers that spontaneously self-assemble during their synthesis due to the solvophobic nature of the growing polymer block [27,28]. Importantly, PISA enables reproducible access to a diverse set of block copolymer nanoparticles of controllable morphology, with spherical micelles, worm-like (or cylindrical) micelles, and vesicles being the most commonly encountered assemblies. Furthermore, PISA is extremely versatile: numerous monomer classes can be polymerized via common controlled/living polymerization techniques [29] in a range of polymerization media, such as water [23] and organic solvents [24]. These undoubted advantages of the PISA process have been utilized to develop polymeric formulations for various applications, including drug delivery [30,31], cell/organelle mimicry [32,33,34], oil additives for friction [35] and viscosity [36,37] modification, chemical sensing [38,39], latex-based films [40], Pickering emulsifiers [41,42], cryopreservation [43,44], and stem cell storage [45], among others. Recently, the generality and versatility of PISA have been further demonstrated and have been used for the preparation of protein-, (poly)peptide-, and amino acid-based nanostructures. The main characteristics of these biomimetic formulations and their great potential in a range of biotechnological and biomedical applications will be discussed in detail in this review article.

## 2. Protein–Polymer Hybrid Nanostructures via PISA

Over the last few years, amphiphilic protein–polymer conjugates have received considerable attention for use as a new class of nanocarriers for drug delivery [13], bioimaging agents [46], and protein therapeutics [47] due to their exceptional degradability [48] and biocompatibility [49]. Some native proteins display undesirable properties, such as reduced stability under non-physiological conditions and enhanced susceptibility to enzymatic degradation, thus giving rise to issues such as unfavorable immunogenic reactions [50]. Protein–polymer conjugates address these issues by providing improved properties; both components render beneficial characteristics to the resulting conjugate (i.e., proteins offer good bio-functionality, whereas polymers provide increased solubility and stability, as well as improved circulation half-life and biodistribution) [51]. Hydrophilic proteins, such as bovine serum albumin (BSA) and human serum albumin (HSA), have been typically selected as the corona-forming component for preparation of amphiphilic protein–polymer conjugates due to being abundantly present in living organisms and easy to isolate and modify [13,52,53]. PISA has recently emerged as an efficient, facile strategy for generating such protein–polymer hybrid nanostructures. PISA can be combined with a “grafting-from” approach to synthesize protein–polymer conjugates, where a hydrophobic polymer is formed from the surface of a hydrophilic, functionalized protein to generate amphiphilic nano-objects that can encapsulate different (bio)molecules in situ, an advantageous property for biomedical applications, such as drug and protein delivery.

Le Droumaguet and Velonia first reported the in situ preparation of bovine serum albumin-*graft*-polystyrene (BSA-*g*-PS) giant amphiphiles via an efficient protein-initiated atom transfer radical polymerization (ATRP)-mediated PISA approach in aqueous solution without the requirement for an organic co-solvent, which would typically result in protein denaturation [54]. A maleimido-capped ATRP initiator was conjugated to BSA to form a BSA-based macroinitiator, which was used as the corona-forming block in the subsequent ATRP-mediated “grafting-from” emulsion polymerization of styrene in phosphate-buffered solution (PBS) (pH = 7.4) to form BSA-*g*-PS conjugate nanoparticles, where PS was the core-forming block. This reaction was carried out both in the presence and in the absence of dimethyl sulfoxide (DMSO) in two separate occasions. Here, spherical aggregates with diameters ranging from 20 to 100 nm were formed, which were used as nanocarriers for the encapsulation of various enzymes. The versatility of this method was further demonstrated using other hydrophilic proteins, such as HSA and reduced human calcitonin. The synthesis of BSA-*g*-PS spherical nanostructures was also reported by Theodorou et al. in what was the first oxygen-tolerant, photoinduced synthesis of protein–polymer bioconjugates via an ATRP “grafting-from” approach (*λ* = 365 nm) in water or PBS, utilizing a similar BSA-based macroinitiator [55]. This multifaceted method was shown to be compatible with several proteins, including, but not limited to, HSA and glucose oxidase (GOx), both of which formed spherical micelles, and four different monomer classes: acrylates, acrylamides, styrenics, and methacrylates. Spherical micelles were also observed for a BSA-*graft*-poly(2-(dimethylamino)ethyl methacrylate) (BSA-*g*-PDMAEMA) conjugate. Importantly, this process did not disrupt the secondary structure of the protein and required low amounts of catalyst. In a more recent report by the same group, the universal character of this aqueous ATRP-mediated photo-PISA procedure was further exploited for the synthesis of amphiphilic BSA-based spherical nano-objects utilizing a wide range of acrylic-, methacrylic-, and acrylamide-based core-forming monomers [56].

**Figure 1:**
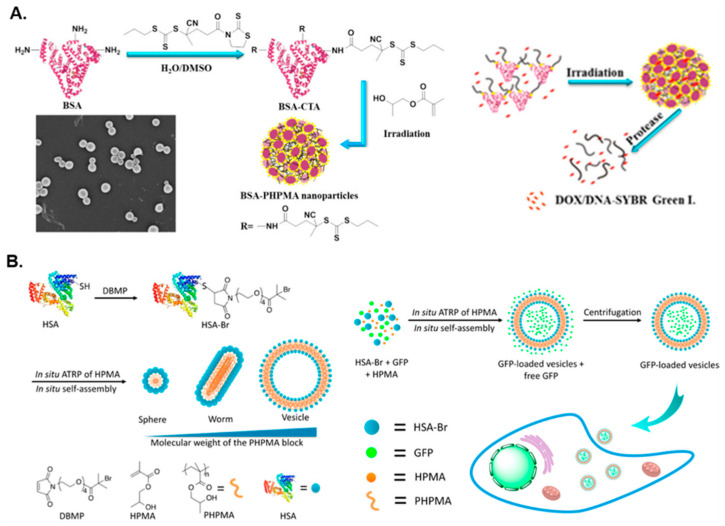
(**A**) Synthesis of BSA-*g*-PHPMA hybrid nano-objects via aqueous RAFT-mediated photo-PISA and subsequent in situ encapsulation of DOX and DNA. Adapted with permission from reference [57]. Copyright 2017 American Chemical Society. (**B**) Synthesis of HSA-*g*-PHPMA hybrid nano-objects via aqueous ATRP-mediated PISA and subsequent in situ encapsulation of green fluorescence protein (GFP) to form GFP-loaded vesicles. Reproduced with permission from reference [58]. Copyright 2017 American Chemical Society.

In addition to the preparation of BSA–polymer hybrid assemblies via ATRP-mediated PISA, BSA has been utilized as a macromolecular chain transfer agent (macro-CTA) for reversible addition–fragmentation chain transfer (RAFT)-mediated PISA syntheses of amphiphilic protein–polymer conjugates. Ma et al. reported an efficient way to synthesize giant protein–polymer amphiphiles [57]. In this approach, a star BSA-based macro-CTA was first synthesized in a water/DMSO mixture and was subsequently chain-extended with 2-hydroxypropyl methacrylate (HPMA) via aqueous photoinitiated RAFT-PISA at room temperature using a blue light source (*λ* = 460 nm) (Figure 1A). The nanoparticle synthesis was conducted at both 5 and 15 wt% solids content using different BSA macro-CTAs, both of which yielded spherical nanostructures of approximately 200–250 nm in diameter. The nanostructures generated at 5 wt% were further used to encapsulate doxorubicin (DOX), Nile red, or deoxyribonucleic acid (DNA), with subsequent controlled protease-mediated release demonstrating the potential of using this type of nanostructures in biomedicine. Notably, encapsulation efficiencies of DOX and DNA reached 13.5% and 11.6%, respectively.

As well as using BSA, a number of research groups have utilized HSA in the development of protein-based nanostructures via PISA. Gao and coworkers prepared HSA-*g*-PHPMA conjugates with varying degrees of polymerization (DPs) of the core-forming PHPMA block in order to tune the morphology of the developed nanostructures [58]. The conjugates were formed through ATRP-mediated dispersion polymerization of HPMA in PBS at 0 °C using an HSA-based macroinitiator. As the HPMA/HSA molar ratio was increased, higher-order morphologies were accessed (i.e., the HSA-*g*-PHPMA nanoparticle morphology transformed from spheres, to worms, to vesicles). Moreover, HSA-*g*-PHPMA vesicles were shown to successfully encapsulate green fluorescence protein (GFP) with an encapsulation efficiency of 11.7% (Figure 1B). An increased cellular fluorescence intensity in the GFP-loaded vesicles relative to free GFP was observed, demonstrating the nanostructures’ practicality and potential use in protein/drug delivery and molecular imaging applications. HSA has also been used to develop pH-responsive protein–polymer hybrid nanostructures. Li et al. described the formulation of HSA-*graft*-poly(2-(diisopropylamino)ethyl methacrylate) (HSA-*g*-PDPA) conjugates via an aqueous ATRP-mediated “grafting-from” PISA approach using an HSA-Br macroinitiator that, with increasing PDPA/HSA molar ratio, yielded morphologies that transformed from spheres, to worms, to aggregates, and finally to spherical vesicles [46]. Dispersion PISA for the synthesis of the conjugate was allowed to progress overnight in PBS at 0 °C. When loaded with indocyanine green (ICG) to form HSA-*g*-PDPA/ICG nanoprobes, these nanoparticles were utilized as effective tumor imaging agents that may offer enhanced precision for tumor removal.

Other antitumor agents have also been developed using similar PISA methodologies. For instance, the Gao group reported the construction of amphiphilic interferon-α-*graft*-(poly((oligoethylene glycol) methyl ether methacrylate)-*block*-PHPMA) (IFN-*g*-(POEGMA-*b*-PHPMA)) conjugate micelles via aqueous ATRP-mediated PISA [47]. The spherical micelles, at 65 nm diameter, were larger than both IFN and IFN-*g*-POEGMA by a factor of 28 and 9.4, respectively. Interestingly, the spherical micelles completely suppressed tumor growth in a mouse model and had a much higher (up to a factor of 21.5) in vitro bioactivity than PEGylated interferon-α PEGASYS, an FDA-approved drug.

**Figure 2:**
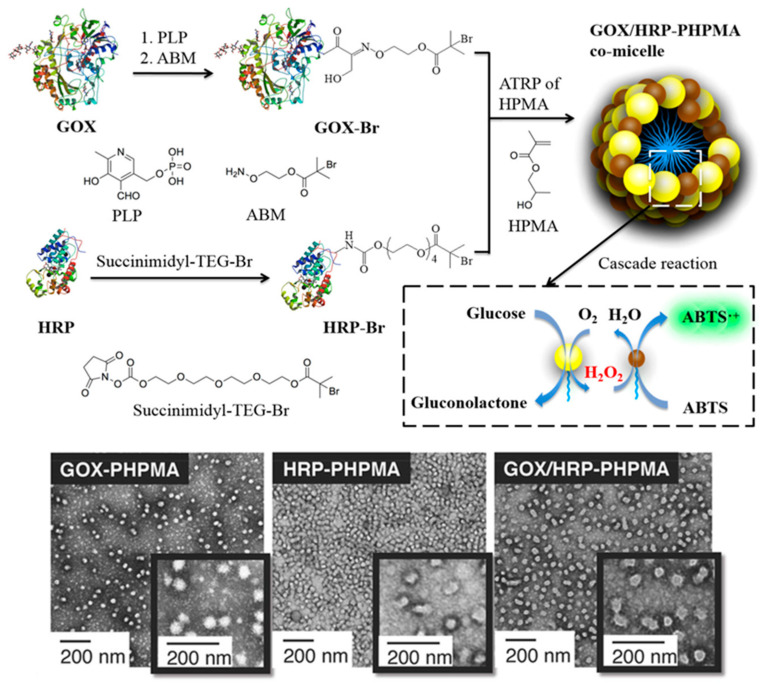
Synthesis of GOx/HRP-*g*-PHPMA hybrid co-micelles via aqueous ATRP-mediated PICA (**top**), and representative TEM images of developed GOx-, HRP-, and GOx/HRP-based spherical nanoparticles (**bottom**). Adapted with permission from reference [60]. Copyright 2019 American Chemical Society.

More recently, Savin and coworkers reported the preparation of biohybrid protein–polymer nanoparticles via aqueous photoinduced ATRP-mediated PISA at *λ* = 440–450 nm (blue light) in PBS using a “grafting-from” approach that utilized a water-soluble superfolder green fluorescent protein (sfGFP) with a genetically encoded ATRP initiator [59]. This sfGFP was selected as the steric stabilizer block due to its structural stability, as a result of optimally designed mutations. Specifically, spherical sfGFP-*g*-PHPMA micelles were produced using this strategy as confirmed by transmission electron microscopy (TEM) and dynamic light scattering (DLS) analyses. As well as being employed as antitumor agents, PISA-derived formulations involving PHPMA cores have been used to develop conjugates for other biomedical applications, such as artificial enzyme complexes. Chiang et al. described a polymerization-induced coassembly (PICA) process to form enzyme–polymer hybrid nano-objects [60]. This was proposed as a novel, controlled method for the design of artificial enzyme complexes, which ultimately did not adversely affect the secondary structure of the proteins. In this study, PICA was carried out using a model GOx/horseradish peroxidase (HRP) system to achieve cascade activity in enzyme complexes. In this ATRP-mediated PICA process, both enzymes were first used to form hydrophilic macroinitiators and then employed to facilitate the in situ growth of PHPMA in PBS at 0 °C to yield amphiphilic conjugates that self-assembled into spherical co-micelles (Figure 2). It was found that the enzyme cascade reaction activities of the co-micelles were much higher (up to a factor of 4.9) than that of the free enzyme mixtures. Interestingly, this enabled the faster detection of glucose over a much broader range of concentrations in comparison with a commercially available glucose assay kit.

In a different approach, Bao et al. reported the synthesis of a myriad of lipase–polymer conjugates via Cu(0)-mediated reversible-deactivation radical polymerization (RDRP) of several monomers with differing hydrophilic/hydrophobic properties [61]. In this work, a *Candida antarctica* lipase B (CALB) macroinitiator was initially synthesized to facilitate the Cu(0)-RDRP of acrylamide and acrylate monomers, including *N*-isopropylacrylamide (NIPAAm), *N*-*tert*-butylacrylamide (tBAm), and methyl acrylate (MA), among others. In the case of hydrophobic monomers, the developed protein–polymer conjugates were able to spontaneously self-assemble into spherical nanostructures in a water/methanol mixture, where the CALB-based macroinitiator was the corona-forming block. The enzymatic activity of the lipase–polymer conjugates was subsequently measured using UV–VIS spectroscopy, demonstrating that in most cases the lipase–polymer conjugates displayed a higher enzyme activity than their native lipase counterpart, as such, highlighting the potential of this PISA method to develop protein–based nanostructures for nanoreactor engineering and enzyme immobilization applications. Table 1 summarizes the main features and breadth of applications of reported protein–polymer hybrid nanostructures prepared by PISA.

## 3. Peptide–Polymer Hybrid Nanostructures via PISA

Peptide–polymer conjugates and hybrid nano-objects from self-assembled peptide–polymer amphiphiles provide a means to combine the robust nature and diverse functionality of polymers with the important biological properties of natural and synthetic oligopeptides [62,63]. Importantly, peptide–polymer hybrid nanostructures have been of significant biomedical interest due to their stimulus-responsive behavior, particularly in the presence of specific enzymes, improved stability and biocompatibility [64,65,66]. Indeed, enzyme-responsive peptide-containing block copolymer nanoparticles have shown great promise in therapeutic formulations and enhanced bioimaging for severe conditions, such as heart disease [67] and cancer [68,69,70]. PISA provides a convenient and scalable route to functional amphiphilic block copolymer nano-objects and, thus, offers an attractive synthetic approach to such responsive peptide–polymer hybrid nanoparticles.

In 2016, Convertine and coworkers were the first to utilize a peptide-functional macromonomer that formed a solvophobic block during PISA [71]. First, the stabilizer block was synthesized via RAFT copolymerization of 2-hydroxyethyl methacrylate (HEMA) and poly(ethylene glycol) methyl ether methacrylate (O300) prior to the RAFT dispersion copolymerization of a peptide-modified methacrylamide macromonomer (MAm-AhxWSGPGVWGASVK) with a zwitterionic sulfobetaine (2-(*N*-3-sulfopropyl-*N,N*-dimethyl ammonium)ethyl methacrylate, DMAPS) in acetic acid at 70 °C, which yielded kinetically trapped spherical micelles.

**Figure 3:**
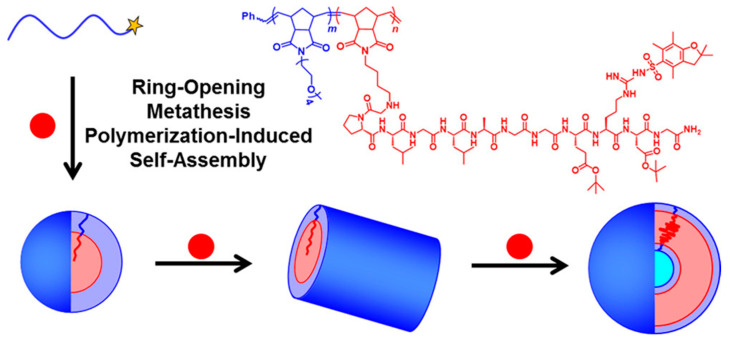
Schematic diagram of ROMPISA conducted using an oligo(ethylene glycol)-based PNB stabilizer block (PNB-OEG, blue) and a peptide-based core-forming norbornene macromonomer (NB-GPLGLAGGERDG, red). Reproduced with permission from reference [72]. Copyright 2017 American Chemical Society.

Subsequently, Gianneschi’s group developed diblock copolymer formulations that utilized pure oligopeptide-based solvophilic stabilizer or solvophobic structure-directing blocks by ring-opening metathesis polymerization-induced self-assembly (ROMPISA) [72,73]. Their initial peptide–polymer conjugate formulation employed room-temperature ROMPISA using Grubbs third generation catalyst and norbornenyl (NB) monomers in *N,N*-dimethylformamide (DMF)/methanol mixtures [72]. Specifically, spherical, wormlike, and vesicular nano-objects composed of diblock copolymer amphiphiles containing a solvophilic oligo(ethylene glycol)-based PNB stabilizer block (PNB-OEG) and a protected peptide-functionalized PNB-based core-forming block were generated. The peptide-functional norbornene macromonomer contained the amino acid sequence GPLGLAGGERDG (Figure 3).

Later, the same group developed a wholly aqueous ROMPISA methodology [74], whereby employing a water-soluble Hoveyda–Grubbs second-generation catalyst enabled PISA to be conducted directly in aqueous media at room temperature without the requirement of oxygen removal (i.e., open-to-air). This allowed for the synthesis of peptide–polymer hybrid nanostructures with coronal peptide functionality by utilizing a hydrophilic stabilizer block formed of a 15-peptide-modified norbornene dicarboximide with the sequence GPLGLAGGWGERDGS, where the core-forming monomer was a quaternary amine-based phenyl norbornene dicarboximide (NB-APh) [73]. Here, spheres and so-called framboidal vesicles were obtained that exhibited enzyme-responsive aggregation on the addition of the proteolytic enzyme thermolysin, which may render these nanomaterials useful as delivery scaffolds for tissue imaging. Importantly, the peptide functionality was positioned in the corona-forming domain of the nanoparticles to maximize accessibility to cell surface receptors.

**Figure 4:**
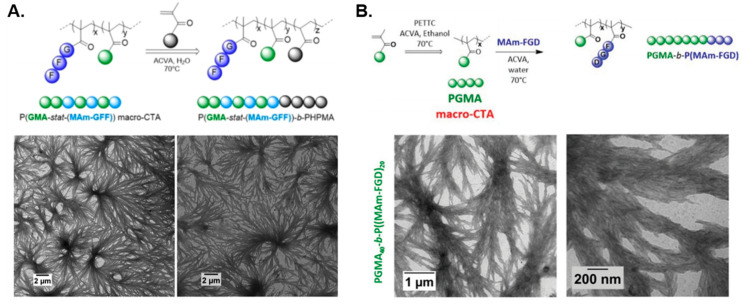
(**A**) Synthesis of P(GMA-*stat*-(MAm-GFF))-*b*-PHPMA block copolymer nanostructures via aqueous RAFT-mediated PISA (**top**), and representative TEM images of developed dendritic fibers (**bottom**). Adapted with permission from reference [77]. Copyright 2020 American Chemical Society. (**B**) Synthesis of PGMA-*b*-P(MAm-FGD) block copolymer nanostructures via aqueous RAFT-mediated PISA (**top**), and representative TEM images of prepared dendritic fibers (**bottom**). Adapted with permission from reference [80]. Copyright 2021 Royal Society of Chemistry.

As highlighted above, RAFT is the most common polymerization technique employed to mediate PISA [27,75], which Semsarilar and coworkers utilized in a plethora of studies to prepare oligopeptide-functional hybrid nano-objects via aqueous PISA. In the group’s first report [76], a water-soluble steric stabilizer was initially prepared via conjugation of a chain transfer agent (CTA) and an amino-terminated oligopeptide chain comprising three lysine residues (i.e., KKK). The resulting tripeptide-functional macro-CTA was then chain-extended with water-miscible HPMA at 70 °C to yield aqueous dispersions of spheres, worms, and vesicles upon varying the PHPMA DP and total solids content. Such nanoparticles were subsequently used to engineer antimicrobial thin film membranes that successfully purified water containing *Staphylococcus epidermidis* bacteria. The same group later incorporated peptide-containing methacrylamide units (MAm-GFF) within a hydrophilic poly(glycerol monomethacrylate) (PGMA) corona-forming block to produce peptide-based P(GMA-*stat*-(MAm-GFF))-*b*-PHPMA block copolymer assemblies in situ via aqueous RAFT-mediated dispersion PISA [77]. In this case, dendritic, flower-like fibers were formed in a self-assembly behavior that was predominantly governed by non-covalent interactions between peptide-decorated monomeric units within coronal chains (Figure 4A), similar to that observed in self-assembling peptides (SAPs) [78]. Interestingly, these fibers transformed into worm-like micelles at 70 °C and lower-order spheres at 4 °C.

In both of these RAFT-PISA formulations, the peptide-rich moieties were located within the solvent-accessible stabilizer block, something that Gianneschi and collaborators further emulated in a room-temperature RAFT-mediated photo-PISA method in acetate buffer (pH = 5.0) [79]. In this study, a peptide-functionalized acrylamide-based macromonomer (Am-KLAKLAKKLAKLAK) was first copolymerized with *N,N*-dimethylacrylamide (DMA) via photoinitiated RAFT-mediated polymerization (*λ* = 450 nm) to form a hydrophilic P(DMA-*stat*-(Am-KLAKLAKKLAKLAK)) macro-CTA prior to its one-pot chain-extension with DMA and diacetone acrylamide (DAAm) under similar reaction conditions. This approach yielded spherical peptide–polymer conjugate nanoparticles of tunable size containing surface pro-apoptotic “KLA”-type peptides that demonstrated enhanced proteolytic resistance and cellular uptake compared with the oligopeptide alone.

The versatility of RAFT-mediated PISA for the preparation of oligopeptide-based assemblies was further demonstrated by Dao et al. [80], who synthesized nano-objects with complex morphologies whose structure-directing solvophobic blocks comprise purely peptide-functional methacrylamide units. In particular, solvophilic PGMA macro-CTAs were initially used to chain-extend the core-forming MAm-GFF methacrylamide macromonomer by ethanolic emulsion PISA to yield dendritic, micrometer-sized structures akin to SAP-type aggregates. Nevertheless, copolymerizing the less solvophobic HPMA monomer within the core-forming block promoted the formation of colloidally stable short worms and spherical vesicles. An alternative RAFT-PISA approach to produce stable, well-defined P(MAm-GFF)-based nanoparticles (spheres or vesicles) involved the utilization of a water/acetonitrile mixture, which is a good solvent system for the MAm-GFF macromonomer and thus overcomes non-covalent peptide–peptide interactions. In the same work, a second, less hydrophobic peptide-modified MAm macromonomer was also used (MAm-FGD), which facilitated RAFT-mediated dispersion PISA in pure water to produce dendritic fibers similar to those previously reported (Figure 4B) [77]. In these cases, the formation of such complex assemblies was primarily governed by the secondary structure that the utilized peptides adopt in solution. In summary, the main characteristics and application scope of currently reported peptide–polymer hybrid nanostructures prepared by PISA are given in Table 2.

## 4. Polypeptide- and Polypeptoid-Based Nanostructures via PISA

In addition to the solid-phase synthesis of short (oligo)peptides (typically between 3 and 20 amino acid residues) with precision sequence and functionality [81,82], a widely utilized alternative strategy for the preparation of protein-mimicking polymeric materials involves the ring-opening polymerization (ROP) of α-amino acid *N*-carboxyanhydrides (NCAs) for the synthesis of well-defined co-polypept(o)ides of controlled molecular weight and low dispersity [83,84,85]. Similar to proteins, these biocompatible, synthetic macromolecules are able to respond to externally applied stimuli and adopt three-dimensional secondary structures, such as α-helices and β-sheets, that can determine their solution properties and potential biomedical applications [86]. Over the past decade, engineering of amphiphilic polypeptide- and polypeptoid-based nanostructures was primarily achieved through conventional self-assembly techniques in dilute solutions [87,88], whereas in situ fabrication of such assemblies by PISA has only very recently been reported, providing a powerful platform for facile access to hybrid polypeptide/polypeptoid-containing biomaterials.

**Figure 5:**
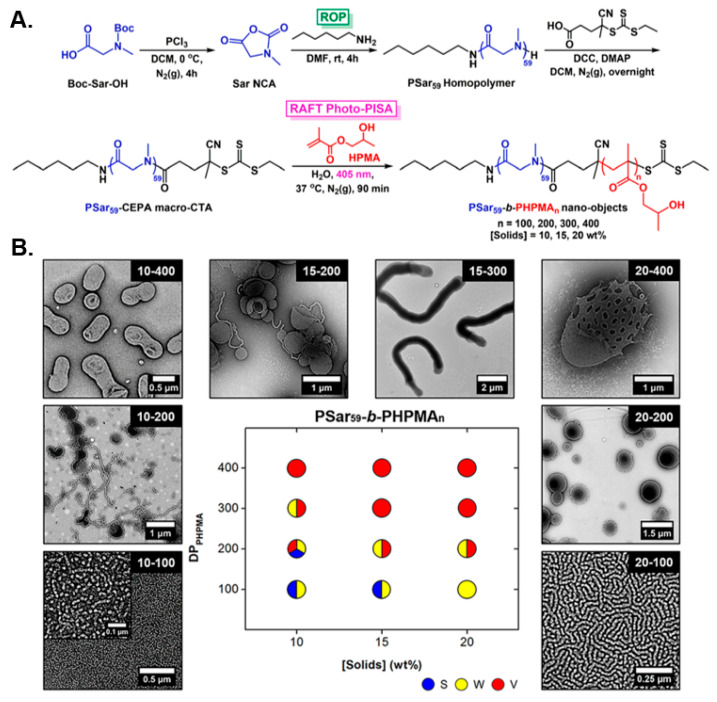
(**A**) Synthesis of PSar-*b*-PHPMA diblock copolymer nano-objects via aqueous RAFT-mediated photo-PISA. (**B**) Phase diagram and representative TEM images for PSar-*b*-PHPMA diblock copolymer nano-objects as a function of total solids concentration and core-block DP. Reproduced with permission from reference [89]. Copyright 2018 American Chemical Society.

In a seminal study, Varlas, O’Reilly, and collaborators reported the development of polypeptoid-based block copolymer nano-objects through the combination of ROP of NCAs and aqueous RAFT-mediated photo-PISA [89]. Specifically, a poly(sarcosine) (PSar) homopolymer was first synthesized by living ROP of sarcosine NCA and was subsequently functionalized with a small-molecule CTA to yield a water-soluble PSar-based macro-CTA. A variety of amphiphilic PSar-*b*-PHPMA diblock copolymer nanostructures were formed following chain-extensions of the developed PSar macro-CTA with HPMA via aqueous photo-PISA (*λ* = 405 nm) under mild reaction conditions (Figure 5A). As typically observed in most PISA formulations, self-assembled morphologies progressed from spheres, to worms, to vesicles in a predictable manner when increasing both the DP of the core-forming PHPMA block and the solids concentration (Figure 5B). Notably, a direct comparison of empty and HRP-loaded PSar-stabilized vesicles with their PEG-based counterparts was also conducted, with their long-term colloidal stability and the ability of such biomimicking nanoreactors to resist proteolytic degradation being evaluated. The PSar-based vesicles exhibited superior properties, highlighting the great potential of PSar as an alternative hydrophilic block to widely employed PEG-based nanomaterials.

In 2019, Yu et al. prepared analogous spherical PSar-*b*-PHPMA block copolymer nanoparticles following a similar two-step approach, involving NCA ROP for the synthesis of a PSar steric stabilizer block and RAFT-mediated dispersion PISA of HPMA in a 2:10 *v*/*v* ethanol/water mixture at 55 °C [90]. In this work, it was crucial that the targeted PHPMA DP was considerably short (≤21 units) in order to minimize the cytotoxicity of the prepared nanoparticles and maintain their size below 200 nm for use as potential drug delivery vehicles. At a next stage, anticancer drug DOX was encapsulated within the solvophobic core of the nanoparticles, and its pH- and temperature-triggered release was assessed, showing that enhanced DOX release was achieved at mildly acidic microenvironments (pH = 5.0) and solution temperatures above 41 °C. The increase in solution temperature also resulted in a noticeable size decrease of the nanoparticles together with their transformation to more anisotropic assemblies. Finally, empty particles were found to be non-cytotoxic against three different breast cancer cell lines, whereas DOX-loaded ones exhibited pronounced cell death capabilities and desired characteristics for controlled drug release applications.

Furthermore, Du and coworkers were the first to develop amphiphilic polypeptide-containing block copolymer nano-objects by ring-opening polymerization-induced self-assembly (ROPISA) of α-amino acid NCAs in organic media [91]. In this report, an amino-terminated poly(ethylene glycol) (PEG-NH_2_) macroinitiator was initially utilized for open-to-air ROPISA of core-forming L-phenylalanine (Phe) NCA in tetrahydrofuran (THF) at low reaction temperature (10 °C), leading to the in situ formation of PEG-*b*-PPhe nanostructures. Upon increasing the Phe NCA/PEG-NH_2_ molar ratio and solids content, morphologies transitioned from kinetically frozen spherical core-shell nanoparticles to larger unilamellar vesicles. Further investigation focusing on the biodegradability of the vesicular assemblies showed that the particles presented good long-term colloidal stability after their post-PISA transfer into aqueous media, while they progressively degraded and disassembled over a 96 h period in the presence of the proteolytic enzyme trypsin. Importantly, the described methodology was also applied for ROPISA of *β*-benzyl L-aspartic acid (BLA) NCA as the core-forming monomer, with the resulting PEG-*b*-PBLA diblock copolymer nano-objects exhibiting similar properties to their PEG-*b*-PPhe analogues.

Recently, Lecommandoux, Bonduelle, and colleagues moved one step further by implementing a similar NCA ROPISA process directly in aqueous media for fabrication of hybrid block copolymer nano-objects with polypeptide-based hydrophobic segments, expanding the biorelevant scope and applicability of the method [92]. Block copolymer amphiphiles were developed via ROPISA of either *γ*-benzyl L-glutamate (BLG) or *N*^ε^-tert-butyloxycarbonyl-L-lysine (*N*BocLys) NCA in an aqueous sodium bicarbonate (NaHCO_3_) solution (pH = 8.5) at 4 °C, using a water-soluble PEG-NH_2_ macroinitiator. The authors exploited the key advantages of PISA, such as the high local monomer concentration within the formed nanoparticle cores linked to fast polymerization kinetics, as well as quantitative conversions to limit the undesired NCA hydrolysis and allow for the construction of a series of self-assembled PEG-*b*-PBLG and PEG-*b*-P(*N*BocLys) nanostructures. Interestingly, elongated needle-like and worm-like micellar morphologies were obtained irrespective of the targeted hydrophobic core-block length and solids content, highlighting the significance of the secondary structure of the polypeptidic domains of the assemblies in directing the extent of chain stretching and regulating the overall ROPISA behavior. The main characteristics and applications of currently reported polypeptide- and polypeptoid-based block copolymer nano-objects prepared by PISA are summarized in Table 3.

## 5. Amino Acid-Based Nanostructures via PISA

Over recent years, a different approach to develop protein/peptide-mimicking synthetic polymers and self-assembled nanostructures, involving the functionalization of vinyl monomers and their corresponding polymers with various amino acid moieties, has attracted particular research interest [21,93,94]. To date, the most commonly utilized strategy encompasses the synthesis of amphiphilic side group-functionalized (co)polymers from amino acid-containing (meth)acrylate/acrylamide-based monomers via a controlled radical polymerization technique, such as ATRP [95,96] and RAFT polymerization [97,98], followed by their self-assembly in aqueous media. Despite the fact that this class of polymers does not adopt secondary structural motifs similar to those encountered in proteins and (poly)peptides (i.e., α-helices or β-sheets), owing to the absence of a polyamide-based backbone, they still possess the ability to undergo reversible conformational changes and self-assembly in response to externally applied stimuli [99,100]. The inherent stimuli-responsiveness of amino acid-based polymers and nanostructures, as well as their interaction capabilities with metal ions and tunable physicochemical properties, have rendered these biomimetic materials excellent candidates for drug delivery, catalysis, and biosensing applications, among others [94,100].

Contrary to the numerous post-polymerization self-assembly methodologies reported thus far for fabrication of amino acid-containing block copolymer nano-objects, PISA has only very recently emerged as an efficient process that enables direct access to such “smart” nanomaterials. In an initial study, Ladmiral, Armes, and coworkers reported the synthesis of water-soluble poly(L-cysteine methacrylate) (PCysMA) and poly(L-glutathione methacrylate) (PGSHMA) macro-CTAs and their subsequent chain-extension via aqueous RAFT-mediated dispersion PISA at 70 °C using HPMA as the core-forming monomer [101]. A series of PCysMA-*b*-PHPMA, PGSHMA-*b*-PHPMA, and (1:9 PCysMA/PGSHMA + PGMA)-*b*-PHPMA diblock copolymer nano-objects were obtained upon varying both the DP of the solvophobic PHPMA block and the total solids content in each case. Higher-order morphologies, such as worm-like micelles and vesicles, could be accessed when PCysMA or binary mixtures of PGMA with either PCysMA or PGSHMA were utilized as the steric stabilizer blocks, whereas only kinetically trapped spherical micelles were developed when using the PGSHMA macro-CTA alone owing to its anionic character. Furthermore, the authors investigated the pH-dependent and thermoresponsive behavior of the worm-like micelles, demonstrating that these nano-objects exhibited positive surface charge at pH < 3.5 and negative coronal charge at pH > 3.5, while they were also able to undergo reversible gelation–degelation on cooling below 4 °C due to a worm-to-sphere morphological transition. It should be noted that the potential redox-responsive character of these nano-objects could not be explored since the free thiol groups of Cys and GSH were utilized as the reactive sites for the preparation of the respective methacrylate monomers by thia-Michael addition.

**Figure 6:**
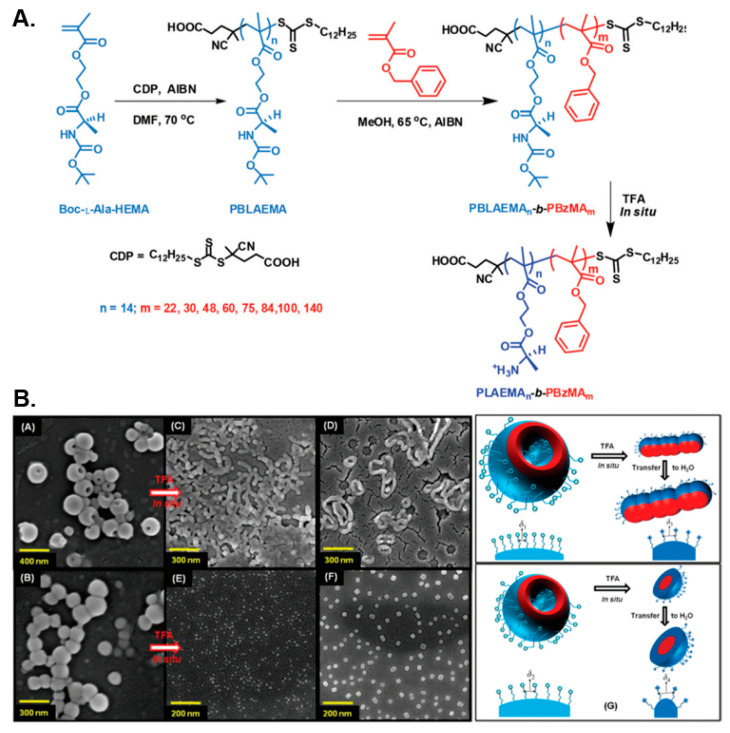
(**A**) Synthesis of PBLAEMA-*b*-PBzMA diblock copolymer nano-objects via alcoholic RAFT-mediated PISA and subsequent Boc-group cleavage for the preparation of respective PLAEMA-*b*-PBzMA nanostructures. (**B**) Representative SEM images of PBLAEMA-*b*-PBzMA vesicles and their corresponding lower-order nano-object morphologies in methanol and water, following in situ Boc-group deprotection of PBLAEMA, along with a schematic illustration of the process. Reproduced with permission from reference [102]. Copyright 2015 Royal Society of Chemistry.

In a similar manner, De and coworkers prepared diblock copolymer nano-assemblies by RAFT-mediated dispersion polymerization of benzyl methacrylate (BzMA) in methanol at 65 °C using Boc-protected poly(L-alanine methacryloyloxyethyl ester) (PBLAEMA) or poly(L-leucine methacryloyloxyethyl ester) (PBLEMA) macro-CTAs in two separate studies [102,103]. In both instances, a diverse set of pure nano-object morphologies, spanning from spherical and worm-like micelles to long fibers and polymersomes, was obtained by systematically varying the length of the core-forming PBzMA block. In the former case, the thermo-reversible gelation–degelation behavior of the formed soft PBLAEMA-stabilized worm gels was also studied, which was shown to be accompanied by a worm-to-sphere transition upon heating the solution from 25 to 65 °C. Moreover, the chiral nature of the nanostructures was demonstrated by circular dichroism spectroscopy, whereas in situ Boc-group deprotection of PBLAEMA units of vesicular formulations resulted in their transition towards lower-order morphologies (i.e., worm-like micelles or spheres) in both alcoholic and acidic aqueous media due to the introduction of cationic primary ammonium moieties within the corona of the particles and the subsequent increase of their interfacial curvature (Figure 6).

In a later report, Chen et al. constructed temperature- and pH-responsive multicompartment block copolymer nanoparticles by RAFT-mediated dispersion PISA, utilizing a dual poly(*N*-acryloylsarcosine methyl ester) (PNASME)/poly(4-vinylpyridine) (P4VP) macro-CTA system for chain-extension of a core-forming styrene monomer in a 80:20 *w*/*w* ethanol/water mixture at 70 °C [104]. Following PISA, the developed AB/CB-type spherical nanostructures were successfully transferred into aqueous milieu via dialysis, whilst maintaining their initial size and shape. Upon exploiting the thermo-sensitive character of PNASME, the ability of its terminal ester groups to be hydrolyzed in alkaline aqueous solutions to yield poly(*N*-acryloylsarcosine) (PNAS), as well as the pH-dependent properties of P4VP, multicompartment nano-objects with tunable size and surface topology were accessed. In particular, fully extended P4VP and PNASME coronal chains were formed in acidic solutions (pH = 2.0) at room temperature, whereas these stabilizing blocks transitioned into their collapsed state at near-neutral pH and elevated temperatures (85 °C), respectively. Combinations of pH and temperature variations, along with ester group hydrolysis of PNASME for PNAS synthesis, resulted in a wide range of core-shell nanoparticles with intriguing microdomain characteristics.

More recently, Zhao et al. prepared thermoresponsive polyion complex (PIC) nanostructures through polymerization-induced electrostatic self-assembly (PIESA) of a cationic arginine-like core-forming monomer, namely, *N*-(2-guanidinoethyl)methacrylamide (GEMA), and simultaneous non-covalent structural locking with anionic poly(2-acrylamido-2-methylpropanesulfonic acid) (PAMPS) by H-bonding [105]. Visible light-initiated RAFT polymerization of GEMA was performed in an acidic aqueous solution of PAMPS at 25 °C using a hydrophilic poly(*N*-2-hydroxypropylmethacrylamide) (PHPMAm) macro-CTA as the corona-forming block. Amphiphilic PIC-based block copolymer nano-objects were developed thatevolved from spheres to worms with an increasing DP of PGEMA. In addition, the authors explored the heating-induced morphological transition of originally prepared nano-objects toward higher-order morphologies, attributed to the dehydration of PHPMAm and increased H-bonding interactions with PGEMA units. This process was shown to be reversible in the case of spherical micelles, formed at PGEMA DP ≤ 30, which were able to evolve to cylindrical structures upon heating to 70 °C, whereas an irreversible worm-to-vesicle transition occurred at a higher PGEMA DP owing to increased electrostatic complexation and effective structural locking. Interestingly, the formation of structurally locked assemblies was further demonstrated by conducting the same PIESA methodology at different reaction temperatures, while always targeting the same core-block DP, revealing that stable higher-order morphologies, such as jellyfish and vesicles, could be obtained by progressively increasing the temperature at which PIESA was conducted.

## 6. Conclusions and Outlook

In recent years, PISA has attracted immense research interest for the development of self-assembled block copolymer nanostructures for various applications owing to its numerous advantages over conventional self-assembly techniques. Consequently, this has also resulted in the emergence of PISA as a highly efficient platform for the preparation of advanced protein-inspired nanomaterials with biotechnological and biomedical scope. In particular, an increasing number of PISA studies have focused on protein–polymer hybrid nano-objects, whereby hydrophilic proteins/enzymes are typically functionalized with radical polymerization-initiating moieties, followed by their chain-extension with selected monomers known to undergo PISA in aqueous media. Expectedly, only kinetically trapped spherical particles were accessed in the majority of these reports due to the large molecular weight of protein-based stabilizer blocks that limit morphological evolution towards higher-order assemblies. Furthermore, PISA has been exploited for the formulation of peptide–polymer hybrid nanostructures of common morphologies, such as spheres, worms and vesicles, but also assemblies of higher complexity, such as dendritic fibers. In these examples, both corona- and core-forming macromonomers bearing oligopeptide functionalities were prepared through solid-phase peptide synthesis and subsequent modification with (meth)acrylamide or norbornene groups for their use in RAFT- or ROMP-mediated PISA, respectively. Importantly, there also exist a limited number of reports on protein-mimicking block copolymer nano-objects prepared via PISA of purely synthetic polypeptide- and amino acid-based amphiphiles. In the former case, the inherent limitations associated with ROP, such as the stringent reaction conditions and specialty equipment required, need to be overcome in future studies in order to increase the practicality of ROPISA as a viable strategy for the synthesis of polypept(o)ide-based nanoparticles. In the latter instance, existing reports were centered on investigating the stimuli-responsiveness of amino acid-containing assemblies on a more fundamental level with no real-time applications shown for these nanomaterials. Ultimately, it is evident that the full potential of PISA for construction of protein-, (poly)peptide-, and amino acid-based nanostructures has yet to be explored. We believe that future research in this field will primarily focus on understanding the effect of secondary structure-adopting (poly)peptides on the chain packing and morphology of obtained nano-objects, as well as engineering stimuli-responsive block copolymer nanoparticles with “on-demand” shape-shifting and cargo-release capabilities for therapeutic applications. Moreover, future in vitro and in vivo investigations utilizing such nanomaterials are expected to reveal their biocompatible and biodegradable character, whilst industrially viable scale-up procedures will need to be developed with the end target of their commercial and clinical use.

## Figures and Tables

**Table 1:** Summary of corona- and core-forming blocks, reaction solvents, obtained morphologies, and applications of protein–polymer hybrid nanostructures prepared via PISA.

Corona-Forming Block	Core-Forming Block	PISA Solvent	Morphologies ^1^	Application	Ref.
BSA, HSA, reduced human calcitonin	PS	PBS (w. DMSO in some cases)	S	Nanoreactors	[54]
BSA, HSA, GOx, β-galactosidase	PS, PDMAEMA, substituted styrenes	PBS, water	S	Nanoreactors	[55]
BSA	MA, MMA, tBA, tBMA	Water	S	-	[56]
BSA	PHPMA	Water	S	Drug delivery	[57]
HSA	PHPMA	PBS	S/W/V	Protein delivery	[58]
HSA	PDPA	PBS	S/W/A/V	Bioimaging	[46]
IFN-*g*-POEGMA	PHPMA	PBS	S	Antitumor agents	[47]
sfGFP	PHPMA	PBS	S	-	[59]
GOx/HRP	PHPMA	PBS	S	Glucose detection	[60]
CALB	NIPAAm, tBAm, MA	Water/methanol	S	-	[61]

^1^ Key: S—spherical micelles, W—worm-like micelles, V—vesicles, A—aggregates.

**Table 2:** Summary of corona- and core-forming blocks, reaction solvents, obtained morphologies, and applications of peptide–polymer hybrid nanostructures prepared via PISA.

Corona-Forming Block	Core-Forming Block	PISA Solvent	Morphologies ^1^	Application	Ref.
P(HEMA-*stat*-O300)	P(DMAPS-*stat*-(MAm-AhxWSGPGVWGASVK))	Acetic acid	S	-	[71]
PNB-OEG	P(NB-GPLGLAGGERDG)	DMF/methanol	S/W/V	-	[72]
P(NB-GPLGLAGGWGERDGS)	PNB-APh	Water	S/V	Biomimicry	[73]
KKK	PHPMA	Water	S/W/V	Antibacterial membranes	[76]
P(GMA-*stat*-(MAm-GFF))	PHPMA	Water	DF/S ^2^/W ^3^	-	[77]
P(DMA-*stat*-(Am-KLAKLAKKLAKLAK))	P(DMA-*stat*-DAAm)	Acetate buffer	S	Therapeutics/biomimicry	[79]
PGMA	P((MAm-GFF)-*stat*-HPMA)	Ethanol	W/V	-	[80]
PGMA	P(MAm-GFF)	Water/acetonitrile	S/V	-	[80]
PGMA	P(MAm-FGD)	Water	DF	-	[80]

^1^ Key: S—spherical micelles, W—worm-like micelles, V—vesicles, DF—dendritic fibers. ^2^ Formed on cooling to 4 °C. ^3^ Formed on heating to 70 °C.

**Table 3:** Summary of corona- and core-forming blocks, reaction solvents, obtained morphologies, and applications of polypeptide- and polypeptoid-based nanostructures prepared via PISA.

Corona-Forming Block	Core-Forming Block	PISA Solvent	Morphologies ^1^	Application	Ref.
PSar	PHPMA	Water	S/W/V	Nanoreactors/biomimicry	[89]
PSar	PHPMA	Ethanol/water	S	Drug delivery	[90]
PEG	PPhe, PBLA	THF	S/V	-	[91]
PEG	PBLG, P(*N*BocLys)	aq. NaHCO_3_	W	-	[92]

^1^ Key: S—spherical micelles, W—worm-like micelles, V—vesicles.
